# Adverse drug reactions and off-label and unlicensed medicines in children: a nested case?control study of inpatients in a pediatric hospital

**DOI:** 10.1186/1741-7015-11-238

**Published:** 2013-11-07

**Authors:** Jennifer R Bellis, Jamie J Kirkham, Signe Thiesen, Elizabeth J Conroy, Louise E Bracken, Helena L Mannix, Kim A Bird, Jennifer C Duncan, Matthew Peak, Mark A Turner, Rosalind L Smyth, Anthony J Nunn, Munir Pirmohamed

**Affiliations:** 1 Department of Women?s and Children?s Health, University of Liverpool, First Floor, Liverpool Women's Hospital, Crown Street, Liverpool L8 7SS, UK; 2 Department of Biostatistics, University of Liverpool, Shelley's Cottage, Brownlow Street, Liverpool L69 3GS, UK; 3 Research and Development, Alder Hey Children?s NHS Foundation Trust, Eaton Road, Liverpool L12 2AP, UK; 4 Oncology Unit, Alder Hey Children?s NHS Foundation Trust, Eaton Road, Liverpool L12 2AP, UK; 5 Institute of Child Health, University College London, 30 Guilford Street, London WC1N 1EH, UK; 6 Department of Molecular and Clinical Pharmacology, University of Liverpool, Ashton Street, Liverpool L69 3GE, UK

**Keywords:** Adverse drug reactions, Pediatrics, Prescribing, Off-label, Unlicensed

## Abstract

**Background:**

Off-label and unlicensed (OLUL) prescribing has been prevalent in pediatric practice. Using data from a prospective cohort study of adverse drug reactions (ADRs) among pediatric inpatients, we aimed to test the hypothesis that OLUL status is a risk factor for ADRs.

**Methods:**

A nested case?control study was conducted within a prospective cohort study. Details of all medicines administered were recorded, including information about OLUL status. The odds ratio for OLUL medicines being implicated in a probable or definite ADR was calculated. A multivariate Cox proportional hazards regression model was fitted to the data to assess the influence that OLUL medicine use had on the hazard of an ADR occurring.

**Results:**

A total of 10,699 medicine courses were administered to 1,388 patients. The odds ratio (OR) of an OLUL medicine being implicated in an ADR compared with an authorized medicine was 2.25 (95% confidence interval (CI) 1.95 to 2.59). Medicines licensed in children but given to a child below the minimum age or weight had the greatest odds of being implicated in an ADR (19% of courses in this category were implicated, OR 3.54 (95% CI 2.82 to 4.44). Each additional OLUL medicine given significantly increased the hazard of an ADR (hazard ratio (HR) 1.3 95% CI 1.2 to 1.3, *P* <0.001). Each additional authorized medicine given also significantly increased the hazard (HR 1.2 95% CI 1.2 to 1.3, *P* <0.001).

**Conclusions:**

OLUL medicines are more likely to be implicated in an ADR than authorized medicines. The number of medicines administered is a risk factor for ADRs highlighting the need to use the lowest number of medicines, at the lowest dose for the shortest period, with continual vigilance by prescribers, in order to reduce the risk of ADRs.

## Background

The licensing of a new drug by regulatory authorities such as the European Medicines Agency is based on the quality, safety and efficacy of the medicine. If the manufacturer satisfies these criteria, the drug will be granted a marketing authorization (MA). The MA sets out terms of use for the medicine. However, this does not preclude the use of the medicine outside of those terms which is known as off-label prescribing. Additionally, clinicians can also prescribe unlicensed medicines, that is, where the drug does not have a MA in their country. In some cases there is a logical basis to the prescription of the drug off-label. In pediatric practice, off-label and unlicensed (OLUL) prescribing has been prevalent because of the lack of assessment of the use of drugs in children during the drug development process [1]. The reported incidence of OLUL use of medicines in children ranges from 36% to 100% [2]. Although the recently introduced pediatric regulation in the EU [3] and the Pediatric Research Equity Act of 2003 in the US [4] are likely to improve the situation, it is going to take time.

The use of OLUL medicines in children is a potential risk factor for adverse drug reactions (ADRs). For instance, in a study of 936 patients, Turner *et al*. found that the proportion of OLUL medicines administered to pediatric inpatients was significantly associated with ADR risk [5]. However, this has not been demonstrated by all studies, many of which were small, employed different methodologies and used inexact and varying definitions. Thus, there is a need for further research in this area [6].

Using the dataset generated by our study evaluating the incidence of ADRs in pediatric inpatients (see accompanying paper), we have employed a nested case?control design to assess the impact of OLUL prescribing on ADR risk in pediatric inpatients. We hypothesized that OLUL prescribing was a risk factor for ADRs in pediatric inpatients.

## Methods

### Selection of cases and controls

We have utilized a prospective cohort study design, undertaken between 1 October 2009 and 30 September 2010, to determine the burden of ADRs in pediatric inpatients (see accompanying paper). The prospective cohort included all inpatients 0 to 16 years old, with the exception of patients in the operating theater, recovery or in the department of radiology, or in the pediatric intensive care unit (PICU) for the entire duration of their admission. Since some patients had multiple admissions over the study period, the cases for the nested case?control study were children on their first admission who had experienced at least one probable or definite ADR (n = 694). These cases were matched 1:1 to controls who were children on their first admission who had not experienced any probable or definite ADRs. Matching was based on the closest date and time of admission, which were chosen in order to ensure that we could assess the relevance of factors such as age and gender as predictors for the occurrence of ADRs. A nested case-control design was chosen because it was not possible to follow up all patients across all their admissions (n = 6,601) in the prospective cohort study and the design allowed us to assess carefully the drugs prescribed and whether those drugs were prescribed within the MA, in both cases and controls.

### Data collection

Detailed information about each patient was recorded as described in the accompanying paper**.** This included the name, route, dose and frequency of all medicines administered and the indication where this was thought to need clarification. A medicine course was defined as the administration of one type of medicine at least once during the admission; this encompassed regular medicines (for example, daily anti-epileptic treatment), short courses (for example, antibiotics) and intermittent doses (for example, paracetamol given as required). Suspected ADRs to medicines administered were recorded and assessed in detail and causality assessment was undertaken using the Liverpool Causality Assessment Tool (LCAT) [7]. If an ADR was found to be probable or definite, the suspected medicine course(s) were then referred to as 'implicated? in an ADR. For each of the 1,388 patients, their record of medicines administered was updated to include a detailed off-label or unlicensed category for each medicine on each day it was administered. The category was assigned by one researcher (JRB) who discussed these classifications with an experienced pediatric clinical pharmacist (AJN). The prospective cohort study did not record theater medicines unless they were implicated in a suspected ADR; these medicines were, therefore, excluded from the case?control study. There were 30 possible off-label categories and five unlicensed medicine categories (Table?1). Categories for off-label use were allocated for each medicine according to the reason(s) why their use was deemed off-label when compared to the terms of the MA for that medicine. The terms of the MA were found in the Summary of Product Characteristics (SmPC) available online from the Electronic Medicines Compendium [8]. With regard to age, if the SmPC mentioned children, the definition of this was assumed to be 28 days to 18 years (as per the definition in the pediatric regulation [3]). If no specific information pertaining to use in neonates was provided, the use of that medicine in neonates was considered to be off-label. Although it was certainly not the case, it was assumed that all neonates were born at term because gestational age was not recorded in this study. Due to the complex nature of the regimes used to treat malignant disease, the classification of cytotoxic medicines was simplified by consulting the British National Formulary for Children (BNFC) for cytotoxic medicines with a UK MA [9]. If the BNFC monograph stated the relevant indication, it was assumed that the use was authorized. If the BNFC monograph stated 'not licensed in children? the use was considered to be off-label. The implications of dosage form manipulation by parents or nursing staff, such as the crushing of tablets or the addition of licensed medicines to food or drinks for ease of administration, was considered to be outside the scope of our analysis.

**Table 1:** Off-label and unlicensed categories

		**Category**	**Definition**
Off-label medicines	Medicines licensed for use in children	1	Authorized - medicine used within the terms of its marketing authorization
		2	Contraindication exists
		3	Dose greater than recommended
		4	Dose greater than recommended and contraindication exists
		5	Not licensed in child of this age (or child below minimum weight stated)
		6	Not licensed in child of this age and contraindication exists
		7	Not licensed by this route
		8	Not licensed by this route and contraindication exists
		9	Not licensed by this route or in a child of this age
		10	Not licensed by this route or in a child of this age and contraindication exists
		11	Not licensed for this indication
		12	Not licensed for this indication and contraindication exists
		13	Not licensed for this indication or at this dose
		14	Not licensed for this indication or at this dose and contraindication exists
		15	Not licensed for this indication or at this age
		16	Not licensed for this indication or at this age and a contraindication exists
		17	Not licensed for this indication or by this route
		18	Not licensed for this indication or by this route and a contraindication exists
		19	Not licensed for this indication or by this route or at this age
		20	Not licensed for this indication or by this route or at this age and a contraindication exists
	Medicines not licensed for use in children	21	Not licensed for use in children
		22	Not licensed for use in children and a contraindication exists
		23	Not licensed for use in children or in adults by this route
		24	Not licensed for use in children or in adults by this route and a contraindication exists
		25	Not licensed for use in children or in adults for this indication
		26	Not licensed for use in children or in adults for this indication and a contraindication exists
		27	Not licensed for use in children or in adults for this indication or in adults by this route
		28	Not licensed for use in children or in adults for this indication or in adults by this route and a contraindication exists.
Medicines excluded from analysis		29	Category cannot be assigned
		30	Theater medicine
Unlicensed medicines		31	Prepared extemporaneously
		32	Manufactured under a specials manufacturing license
		33	Chemical
		34	Import
		35	Awaiting a MA (for example, previous trial medicine)

### Statistical methods

Odds ratios (OR) with 95% confidence intervals (95% CI) were calculated for all OLUL medicines implicated in a probable or definite ADR. Additionally, ORs with accompanying 95% CI were calculated for each OLUL category implicated in a probable or definite ADR in comparison to authorized medicines.

A Cox proportional hazards regression model for an ADR was fit to the data. Results are given in terms of the hazards ratio (HR) and associated 95% CI. In addition to the number of OLUL medicines, the following risk factors were included in the model: age, gender, having received a general anesthetic (GA), oncology patient status and the number of authorized medicines. Due to clinical importance, all risk factors remained in the final model.

All statistical analysis was carried out using the statistical software package R (version 2.13.2) using a two-sided significance level of 0.05 (5%) throughout.

### Reporting

This study was reported according to the Strengthening the Reporting of Observational Studies in Epidemiology (STROBE) guidelines [10].

### Ethics

This study used routinely collected clinical data in an anonymized format. The Chair of Liverpool Paediatric LREC informed us that this study did not require individual patient consent or review by an Ethics Committee.

## Results

### Participants

Clinical data for 1,388 patients were analyzed throughout their first admission. A total of 694 (50%) were cases; 634 (45.6%) were female; 294 (21.2%) were <1 year old, 341 (24.6%) were 1 to 4 years old, 384 (27.7%) were 5 to 11 years old and 369 (26.6%) were teenagers (>12 years old). The median age was 5.9 years (interquartile range (IQR) 1.4 to 12.4 years). A total of 10,699 drug courses were administered in this study.

### Medicine courses

A total of 10,699 medicine courses were administered to the 1,388 patients included in this study. Within this nested cohort, there were 785 suspected ADRs deemed definite or probable in 694 patients during their first admission. Of the suspected ADRs, 62 (7.9%) were deemed definite and 723 (95.1%) probable. Of these ADRs, 505 (64.3%) involved one medicine course, 172 (21.9%) involved two medicine courses, 77 (9.8%) involved three and 31 (3.9%) involved four or more. Of the 10,699 total medicine courses, 10,145 (94.8%) could be categorized using one of the definitions listed in Table?1. The remaining 554 (5.2%) courses could not be categorized because the prescription record did not provide the required information (for example, missing dose information or insufficient detail about the exact preparation used).

A total of 6,980 (68.8%) of all medicine courses were authorized, 2,407 (23.7%) were off-label and 758 (7.5%) were unlicensed. A total of 435 (6.2%) of all authorized medicine courses were implicated in at least one probable or definite ADR compared with 298 (12.4%) of off-label medicine courses and 113 (14.9%) of unlicensed medicine courses. Comparing the rate of OLUL prescribing among age groups, the neonatal category had the fewest patients (n = 75) but the greatest proportion of OLUL prescriptions (58.9%). The proportion of OLUL prescriptions implicated in at least one ADR was greatest in the 'school-aged? category (17.0% of prescriptions; see Additional file 1). The OR ratio of an OLUL medicine being implicated in an ADR when compared with an authorized medicine course was 2.25 (95% CI 1.95 to 2.59).

In total, 19 of the OLUL categories were utilized. Table?2 shows the number of medicine courses in each of these OLUL categories. Category 11: drug licensed for children but given for a different indication is the most common category of off-label medicine use (n = 764; 31.7%). Categories 3, 5 and 11 together represented 2,050 (85.2%) of all off-label medicine courses. Category 32: manufactured under a specials license was the most common category of unlicensed medicine (n = 577; 76%).

**Table 2:** Total number of medicine courses in each authorized, off-label or unlicensed category and number implicated in at least one PD ADR (n = 10,145)

		**Category**^**a**^	**Number of medicine courses**	**% of courses implicated in at least one PD ADR**	**Odds ratio of ADR versus authorized**	**95% CI**
Off-label medicines	Medicines licensed for use in children	1	6,980	6.2	1.00	-
		2	1	0	-	-
		3	698	2.7	0.42	0.26 to 0.67
		5	588	19.0	3.54	2.82 to 4.44
		6	1	0	-	-
		7	61	9.8	1.64	0.70 to 3.83
		11	764	14.3	2.50	2.00 to 3.13
		13	8	0	-	-
		15	35	25.7	5.21	2.43 to 11.18
		17	21	0	-	-
		19	2	0	-	-
	Medicines not licensed for use in children	21	215	18.6	3.44	2.41 to 4.91
		22	1	100.0	-	-
		23	1	0	-	-
		25	11	18.2	3.34	0.72 to 15.52
Unlicensed medicines		31	143	14.7	2.59	1.61 to 4.16
		32	577	14.9	2.64	2.05 to 3.38
		33	1	0	-	-
		34	37	16.2	2.91	1.21 to 7.02

^a^see Table?1 for category definitions. PD ADR, probable or definite ADR; adverse drug reaction; CI, confidence interval.

Table?2 shows the proportion of medicine courses from each category implicated in at least one probable or definite ADR in comparison to the proportion of authorized medicine courses implicated (n = 6,980; 6.2%). Further analysis was completed on categories that contained >100 medicine courses. Results showed that category 3: medicines licensed for use in children but given at a dose greater than recommended had a lower risk of being implicated in an ADR than category 1: authorized medicines (OR 0.42, 95% CI 0.26 to 0.67). Category 5: medicines licensed in children but given to a child below the minimum age or weight had the greatest risk of being implicated in an ADR (OR 3.54, 95% CI 2.82 to 4.44).

Table?3 shows the proportion of drug courses implicated in a probable or definite ADR specifically for drugs with more than 100 courses administered ? together with the proportion of courses that were categorized as OLUL. Fentanyl via the epidural route had 44.3% of courses implicated with 100% of courses categorized as unlicensed. Fentanyl via other routes had 48.0% of courses implicated in at least one ADR with 99.3% of courses categorized as off-label. Morphine via any route had 35% of courses implicated, of which 50.9% were OLUL. Table?4 shows the four most frequently implicated medicines by OLUL category. The majority of fentanyl courses were category 11: given for a different indication and 88.0% of implicated fentanyl courses fell into this category. These courses were mainly fentanyl administered in nurse- or patient-controlled analgesia pumps for post-operative pain management. Just under two thirds of morphine courses were authorized and 49.1% of implicated morphine courses fell into this category whereas just over a third of morphine courses were category 5: not licensed in a child of this age or a child below the minimum weight stated and 50.3% of implicated morphine courses fell into this category. Since the OLUL use of morphine and fentanyl was common and ADRs to these medicines were also common, we examined the impact of removing these medicines, regardless of category, from our analysis. This left 6,667 authorized courses and 2,712 OLUL courses that were administered; this resulted in at least one ADR associated with 349 authorized and 248 OLUL courses. However, despite this, OLUL medicines were still more likely to be implicated in an ADR than authorized medicines (OR 1.82, 95% CI 1.54 to 2.16).

**Table 3:** Medicines course frequency administered, implicated and off-label, unlicensed or unknown (only medicines with > 100 courses shown, n = 7,007)

	**Medicine (number of courses)**	**Number of courses off-label or unlicensed (%)**^**a**^	**Number of authorized courses implicated in at least one PD ADR (%)**	**Number of off-label or unlicensed courses implicated in at least one PD ADR (%)**	**Number of courses unknown**
Medicines with only authorized courses	Cefuroxime (245)	0 (0%)	15 (6.5%)	-	1
	Cefotaxime (388)	0 (0%)	40 (10.3%)	-	0
Medicines with off-label courses	Chlorphenamine (339)	1 (0.3%)	1 (0.3%)	0	1
	Diazepam (107)	1 (1.9%)	1 (1.0%)	0	0
	Ibuprofen (545)	26 (4.8%)	4 (0.8%)	1 (3.8%)	0
	Lactulose (272)	13 (4.8%)	7 (2.7%)	1 (0.1%)	0
	Cefalexin (148)	9 (6.1%)	8 (5.8%)	1 (11.0%)	0
	Metronidazole (257)	21 (8.2%)	19 (8.0%)	3 (14.3%)	0
	Furosemide (110)	13 (11.8%)	13 (13.4%)	2 (15.4%)	0
	Paracetamol (1786)	596 (33.4%)	1 (0.1%)	0 (0%)	2
	Ondansetron (550)	290 (52.7%)	18 (8.5%)	14 (2.5%)	48
	Salbutamol (146)	84 (57.5%)	3(4.8%)	10 (11.9%)	0
	Ranitidine (109)	65 (59.6%)	1 (2.3%)	0 (0%)	0
	Dexamethasone (166)	107 (64.5%)	7(13.5%)	3 (2.8%)	7
	Fentanyl (150)	149 (99.3%)	0 (0%)	72 (48%)	0
Medicines with unlicensed courses	Fentanyl and Levobupivicaine epidural (106)	106 (100.0%)	-	47 (44.3%)	0
Medicines with off-label and unlicensed courses	Codeine Phosphate (752)	12 (1.6%)	49 (10.1%)	0 (0%)	257
	Morphine (500)	198 (39.8%)	86 (28.5%)	89 (44.9%)	0
	Diclofenac (331)	149 (45.0%)	2 (8.7%)	3 (2.0%)	159

^a^Drugs within each category listed in ascending order of % off-label or unlicensed. PD ADR, probable or definite adverse drug reaction.

**Table 4:** Off-label and unlicensed category proportions for medicines with >10% of courses implicated in at least one PD ADR

**Category**^**a**^	**Fentanyl (implicated)**	**Fentanyl + Levobupivicaine Epidural (implicated)**	**Morphine (implicated)**	**Codeine (implicated)**
1	1 (0)	-	302 (86)	483 (49)
3	-	-	2 (0)	-
5	1 (0)	-	189 (88)	9 (0)
11	136 (66)	-	6 (1)	-
15	12 (6)	-	-	-
29	-	-	-	257 (33)
32	-	106 (47)	1 (0)	3 (0)
Total	150 (72)	106 (47)	500 (175)	752 (99)

^a^see Table?1 for category definitions. PD ADR, probable or definite adverse drug reaction.

### ADR risk factors

Multivariate risk factor analysis of the nested cohort (Table?5) showed that age on admission and receipt of a GA both had a significant effect on ADR risk. Gender and oncology patient status did not have a significant effect on the hazard of an ADR. The hazard of an ADR increased by 30% with each additional OLUL drug given (median daily number of OLUL drugs administered 1; IQR 0 to 2). Similarly, the hazard of an ADR increased by 20% with each additional authorized drug (median daily number of authorized drugs administered 2; IQR 1 to 3).

**Table 5:** ADR risk factors assessed by multivariate analysis

**Covariate**		**HR (95% CI)**	***P*-value**
Gender	Female	1	0.152
	Male	0.896 (0.770 to 1.042)	
Age on admission (years)		1.036 (1.021 to 1.052)	<0.001
Received a GA	No	1	<0.001
	Yes	5.295 (4.417 to 6.349)	
Oncology	No	1	0.655
	Yes	0.926 (0.661 to 1.298)	
Number of authorized medicines		1.217 (1.171 to 1.263)	<0.001
Number of off-label and/or unlicensed medicines		1.267 ( 1.201 to 1.336)	<0.001
Number of uncategorized medicines		1.138 (0.969 to 1.338)	0.116

ADR, adverse drug reaction; CI, confidence interval; GA, general anesthesia; HR, hazard ratio.

## Discussion

In the largest study undertaken so far, we have investigated the role of OLUL medicines in predisposing pediatric inpatients to ADRs. The prevalence of OLUL prescriptions in pediatric inpatients ranges from 18% to 60% and 3.4% to 36%, respectively [2]. The corresponding figures in our dataset were 23.7% and 7.5%, respectively. These are collectively similar to previous UK studies, where combined OLUL prescribing rates of 35% [5] and 30% [11] have been reported. This is perhaps not surprising since many of the medicines used in children are the older generic medicines, which pre-date the introduction of pediatric regulation in 2007 [3]. Our findings, however, contrast with OLUL prescribing rates in other European countries, where rates of up to 66% have been reported [11]. This reflects differences in study settings, definitions of OLUL prescribing and pharmacy practice between countries. With respect to the latter, for example, there is extensive modification and manufacture of medicines by hospital pharmacies in the Netherlands [12]. Our data show that OLUL medicines were significantly more likely to be implicated in an ADR than medicines used within the terms of their MA (OR 2.25, 95% CI 1.95 to 2.59). The risk estimate is higher than that found previously [5,13] which may be a reflection of the fact that the previous studies were smaller (OR 1.5, 95% CI 1.11 to 1.93 and OR 1.08, 95% CI 0.50 to 2.35) [5,13], looked at different ward types (for example, included pediatric intensive care) [5] and used different definitions of OLUL medicines [13]. We also categorized ADR risk according to the type of OLUL medicine use. By focusing on six categories which all had more than 100 medicine courses, we found that (1) medicines licensed for use in children but given at a dose greater than recommended had a lower risk of being implicated in an ADR than authorized medicines; and (2) medicines licensed in children but given to a child below the minimum age or weight had the greatest risk of being implicated in an ADR. These two findings seem counter-intuitive but can be explained by the fact that 69% of the medicine courses given at a higher dose than recommended were paracetamol. This reflects the widespread use of 15 to 20 mg/kg doses for 'severe symptoms? as recommended in the BNFC [9]. Paracetamol at these doses is relatively safe, particularly in in-patient settings and, indeed, paracetamol was rarely implicated in ADRs throughout the entire study (Table?4).

Multivariate analysis indicated that risk factors for ADRs were the administration of a GA and the number of medicines administered per day, consistent with the findings of the cohort study (see accompanying paper). Furthermore, our finding is consistent with that of Santos *et al*. [14] who found that off-label medicine use was significantly associated with ADR risk (RR 2.44, 95% CI 2.12 to 2.89). However, in our study, we have dissected medicine use and show that the number of OLUL medicines administered per day had an influence on ADR risk similar to that of the number of authorized medicines administered per day. Most studies, including those in adults, have shown that ADR risk increases with the number of medicines used by patients, [5,13,15-17] which reflects the complex interaction that occurs between drugs targeting different biological systems within the body, the interaction with disease (that is, sicker patients are more likely to require a higher number of medicines) and the occurrence of drug-drug interactions.

Our findings, which relate to both OLUL and authorized medicines, highlight the need for good prescribing practice in reducing ADRs. Thus, the minimum number of drugs should be given for the treatment of a disease process, at the lowest possible dose for the shortest possible time. Nevertheless, our data implicate OLUL medicines as risk factors for ADRs in pediatric inpatients. Off-label use is complicated and in some cases can be justified by the fact that evidence which may not necessarily have led to a change in the SmPC is available in the scientific literature as a result of academic investigations [18]. For instance, a substantial proportion of off-label prescribing in pediatric oncology may reflect evidence-based protocol recommendations for specific oncological diseases. In contrast, the evidence for the use of some analgesic medicines in children is less robust [1]. Some of the most commonly implicated medicines in our study were frequently used off-label (for example, dexamethasone). However, we have no evidence that if these products were used in accordance with a MA, they would be implicated in any fewer ADRs. An area of concern identified in our data is the use of fentanyl, either when used off-label or unlicensed, where about 48% of courses were implicated in ADRs. A key issue with fentanyl may be the dose used in children, suggesting a need for further evaluation of dosing strategies.

With all drugs, irrespective of their licensing status, the dose administered and, thus, the systemic exposure to that medicine is an important determinant of the likelihood of an ADR. The importance of this is highlighted by our finding that medicines licensed in children but given to a child below the minimum age or weight had the greatest risk of being implicated in an ADR, reflecting the lack of pharmacokinetic data in children of different ages and/or weights. Advances in the development, and application, of pediatric pharmacokinetic models will be important in defining, and implementation of, age- and weight (or body surface area)- specific dosing regimens [19]. While such approaches are now being incorporated in pediatric investigation plans for new medicines, the challenge for all stakeholders will be how to improve this knowledge for medicines already on the market, most of which are not only generic off-patent compounds but are also the most widely used.

Our study has several limitations: we required a minimum amount of information to be available about the use of a medicine before it could be categorized as off-label. The absence of this information was a result of how medicines were recorded on prescription charts; in general, they were prescribed by the name of the active ingredient and details such as the exact preparation administered were not recorded. Hence, the prescription chart records were adequate for their primary purpose but not for our study. We did not consider the implications of dosage form manipulation by parents or nursing staff, but this is a significant aspect of drug administration in pediatrics which does warrant investigation [20]. Further limitations are the assumptions outlined in our methodology which pertain to the SmPC definitions of age, gestational age and the classification of cytotoxic medicines.

## Conclusion

In one of the largest inpatient pediatric studies, we have shown that OLUL prescribing is a risk factor for ADRs and identified some drugs/classes where further work is needed. The reasons for this are complex and varied, and the finding must be put in context of the fact that the number of medicines *per se*, irrespective of the licensing status, is also a risk factor for ADRs. Better dosing schedules for medicines, particularly those with a narrow therapeutic index, are likely to be key in reducing the burden of ADRs in children. Clinicians prescribing for children should be vigilant for the occurrence of ADRs and should prescribe the minimum number of drugs at the lowest possible dose and shortest duration of time, with continual monitoring to stop drugs when relevant and to ensure that ADRs are detected as early as possible.

## Abbreviations

ADRs: Adverse drug reactions; BNFC: British National Formulary for Children; CI: Confidence interval; GA: General anesthesia; HR: Hazard ratio; IQR: Interquartile range; OLUL: Off-label and unlicensed; MA: Marketing authorization; OR: Odds ratio; SmPC: Summary of Product Characteristics.

## Competing interests

RLS and MPir are members of the Commission on Human Medicines. MPir chairs the Pharmacovigilance Expert Advisory Group, while RLS chairs the Paediatric Medicines Expert Advisory Group. RLS and MPir are senior NIHR investigators. The views expressed in this article are solely those of the authors and not of any institutions that they represent. The funders had no role in study design, data collection, analysis, decision to publish, or preparation of the manuscript. The other authors declare they have no conflicts of interest.

## Authors? contributions

MP, AJN, MAT, MPir and RLS planned the study, were involved in study design and critically revised the manuscript. JRB, AJN, MP and JJK designed the study, developed the methodology and the study protocol. JRB, ST, LEB, HLM, KAB and JCD collected data. JJK, EJC and JRB designed the analysis plan. EJC and JRB conducted the analysis. MPir acts as guarantor and accepts responsibility for the integrity of the work as a whole. All authors read and approved the final manuscript.

## Supplementary Material

Additional file 1: **Distribution of authorized, OLUL medicine courses (n = 10,145) by patient age category.** Count of OLUL medicine courses by age category, count of authorized medicine courses implicated in at least one ADR, count of OLUL medicine courses implicated in at least one ADR. Click here for file
