# Supplementary material for: Adverse drug reactions and off-label and unlicensed medicines in children: a nested case?control study of inpatients in a pediatric hospital
Source: BMC Med. 2013 Nov 7;11:238. doi: 10.1186/1741-7015-11-238 (PMC4231613; doi:10.1186/1741-7015-11-238)
Supplement: Additional file 1: — Distribution of authorized, OLUL medicine courses (n = 10,145) by patient age category. Count of OLUL medicine courses by age category, count of authorized medicine courses implicated in at least one ADR, count of OLUL medicine courses implicated in at least one ADR. [file 1741-7015-11-238-S1.pdf]

Distribution of authorised, off-label and unlicensed medicine courses (n = 10 145) by patient age category

Patient age category[§]	Total patients	Total medicine courses	Total OLUL medicine courses	Total authorised medicine courses implicated in at least one ADR	Total OLUL medicine courses implicated in at least one ADR
**Neonate**	75 (5.8%)	440	259 (58.9%)	10 (5.5%)	20 (8%)
**Infant**	230 (17.9%)	1434	441 (30.8%)	52 (5.2%)	41 (9%)
**Pre-school**	390 (30.3%)	2934	772 (26.3%)	109 (5.0%)	107 (14%)
**School-aged**	322 (25.0%)	2539	743 (29.3%)	108 (6.0%)	128 (17%)
**Teenage**	369 (26.6%)	2798	950 (34.0%)	156 (8.4%)	115 (12%)
**Total**	1386[‡]	10145	3165	435	411

[§] Neonate 0-27 days, Infant < 1year, Pre-school 1-5 years, School aged 5-11 years, Teenage > 11 years

[‡] Medicine category unknown for all medicines administered to 2/1388 participants
